# Three fluoroquinolones with different epithelial lining fluid penetration in a murine lung infection model: an experimental site-specific PK/PD study

**DOI:** 10.1128/aac.00375-26

**Published:** 2026-06-22

**Authors:** Ayaka Hosoi, Shaoqing Duan, Sora Shimada, Kenta Suzuki, Yuki Mizukami, Ryuya Shintani, Mishu Takahashi, Natsuki Satake, Takuma Muraishi, Yuko Okamoto, Yuki Igarashi, Yuki Enoki, Kazuaki Taguchi, Kazuaki Matsumoto

**Affiliations:** 1 Division of Pharmacodynamics, Keio University Faculty of Pharmacy, Minato-ku, Tokyo, Japan; 2 Graduate School of Biomedical and Health Sciences, Hiroshima University, Hiroshima, Japan; Columbia University Irving Medical Center, New York, New York, USA

**Keywords:** fluoroquinolones, pharmacokinetics/pharmacodynamics, epithelial lining fluid, murine lung infection model, site-specific drug exposure

## Abstract

Antimicrobial efficacy in pneumonia is strongly influenced by site-specific drug exposure; however, pharmacokinetic/pharmacodynamic (PK/PD) indices based on plasma concentrations may not adequately reflect drug exposure at the pulmonary infection site. This study evaluated how differences in pulmonary distribution influence the magnitude and interpretation of PK/PD targets by comparing three fluoroquinolones with distinct epithelial lining fluid (ELF) penetration profiles. Using a murine *Streptococcus pneumoniae* lung infection model, levofloxacin (LVFX), garenoxacin (GRNX), and lascufloxacin (LSFX) were evaluated under identical experimental conditions. Minimum-inhibitory concentrations (MICs) were determined by broth microdilution. Pharmacokinetics were assessed in plasma and ELF, and pharmacodynamics were quantified as changes in pulmonary bacterial burden. PK/PD relationships and exposure targets were estimated based on plasma- and ELF-derived indices. The MICs of LVFX, GRNX, and LSFX were 0.512, 0.032, and 0.032 mg/L, respectively. In plasma-based PK/PD analyses, GRNX and LSFX exhibited bactericidal effects at lower *f*AUC/MIC values than LVFX. In contrast, ELF-based analyses revealed greater interdrug differences in PK/PD targets attributable to differences in ELF penetration. Notably, LSFX showed substantial divergence between plasma- and ELF-based PK/PD targets, indicating a strong influence of site-specific exposure on antibacterial efficacy. When ELF-based PK/PD targets were translated to predicted human plasma exposure, the resulting values were generally consistent with reported clinical PK/PD benchmarks. Site-specific PK/PD analysis incorporating pulmonary drug distribution is important for interpreting antimicrobial efficacy in pneumonia. For agents with high ELF penetration, ELF-based PK/PD targets may provide a more relevant framework for understanding antibacterial effects and optimizing dosing strategies.

## INTRODUCTION

Site-specific drug exposure is a key determinant of antimicrobial efficacy. In pneumonia, drug exposure at the infection site, namely, the epithelial lining fluid (ELF), is more directly related to antibacterial activity than concentrations in plasma alone (1–3). When antibacterial efficacy is evaluated solely on plasma concentrations, differences in drug penetration into the ELF may be overlooked, potentially leading to misinterpretation of pharmacokinetic/pharmacodynamic (PK/PD) targets and therapeutic outcomes (4, 5). However, limited studies have directly compared the PK/PD characteristics of antimicrobials with differing ELF penetration under identical experimental conditions using the same pneumonia model, evaluating both plasma and ELF concentrations.

Respiratory fluoroquinolones are often discussed as a therapeutic class for the treatment of pneumonia. However, previous studies have reported substantial differences in pulmonary drug distribution among individual agents, particularly in ELF. The clinical and pharmacodynamic implications of these differences remain incompletely understood. Therefore, we focused on fluoroquinolones, a clinically important class for the treatment of respiratory infections with substantial variability in pulmonary distribution. Among the PK/PD indices, the ratio of the area under the concentration–time curve to the minimum-inhibitory concentration (AUC/MIC) has been consistently identified as the primary determinant of fluoroquinolone efficacy (6–8). Levofloxacin (LVFX), garenoxacin (GRNX), and lascufloxacin (LSFX) are fluoroquinolone antimicrobial agents with demonstrated efficacy against respiratory tract infections (9–12). LVFX is widely used in clinical practice and is regarded as a representative comparator for the treatment of respiratory infections (13, 14). In contrast, GRNX and LSFX are newer respiratory fluoroquinolones approved for clinical use in Japan and have been evaluated for the treatment of respiratory tract infections. In humans, GRNX demonstrates moderate ELF penetration, whereas LSFX has been reported to achieve markedly higher ELF exposure (10, 15, 16).

This study evaluated the PK/PD characteristics of LVFX, GRNX, and LSFX in a murine *S. pneumoniae* lung infection model using plasma- and ELF-based PK/PD analyses to clarify how site-specific drug exposure influences PK/PD target interpretation and antibacterial efficacy in pneumococcal pneumonia.

## MATERIALS AND METHODS

### Antimicrobials

LVFX, GRNX, and LSFX were used in this study.

For *in vitro* experiments, LVFX powder was purchased from Tokyo Chemical Industry Co., Ltd. (Tokyo, Japan). For *in vivo* experiments, commercially available LVFX for injection (Cravit, Daiichi Sankyo Co., Ltd., Tokyo, Japan) was used.

GRNX mesilate hydrate and LSFX hydrochloride were provided by Kyorin Pharmaceutical Co., Ltd. (Tokyo, Japan).

Stock solutions for the quantitative analysis were prepared by accurately weighing each antimicrobial agent. LVFX powder and GRNX mesilate hydrate were dissolved in ultrapure water, whereas LSFX hydrochloride was dissolved in methanol to obtain stock solutions.

Antimicrobial stock solutions and working dilutions for *in vitro* studies were prepared according to CLSI guidelines (17, 18). For *in vivo* studies, LVFX was diluted with sterile physiological saline before administration. GRNX and LSFX were weighed accurately and dissolved in sterile ultrapure water immediately before use.

### Bacterial strain

A strain of *S. pneumoniae* (ATCC 49619) was used in this study.

This strain was obtained from the American Type Culture Collection (ATCC, Manassas, VA, USA) and used as a quality control strain for antimicrobial susceptibility testing in accordance with the CLSI guidelines (17).

Frozen stock cultures were sub-cultured onto sheep blood agar plates prior to each experiment. Bacterial cultures were incubated at 37°C in a 5% CO₂ atmosphere overnight, and freshly prepared colonies were used.

### Animals

Five-week-old female ddY mice were purchased from Sankyo Labo Service Corporation (Tokyo, Japan) and used in all experiments. The mice were housed under controlled environmental conditions (12-h light/dark cycle, controlled temperature, and humidity) with free access to food and water. After a 1-week acclimation period, the mice were used for subsequent experiments.

Neutropenia was induced by intraperitoneal administration of cyclophosphamide at 150 mg/kg 4 days and 100 mg/kg 1 day before the infection. On the day of infection, colonies of *S. pneumoniae* were suspended in saline and adjusted to the desired concentrations based on turbidity measurements using a McFarland densitometer. Under general anesthesia, mice were intratracheally inoculated with 20 µL of the bacterial suspension, corresponding to approximately 1 × 10^7^ colony-forming unit (CFU) per mouse, to establish a lung infection. Each experimental group consisted of three mice. All animal experiments were approved by the Keio University Animal Experiment Committee (A2024-012) and conducted in accordance with institutional guidelines for animal care and use.

### *In vitro* susceptibility testing

The MICs of LVFX, GRNX, and LSFX against *S. pneumoniae* ATCC 49619 were determined using the broth microdilution method in accordance with the CLSI guidelines (17, 18).

Two-fold serial dilutions of each antimicrobial agent were prepared in cation-adjusted Mueller–Hinton broth supplemented with 2.5% lysed horse blood. After incubation for 24 h at 37°C in a 5% CO_2_ atmosphere, the MIC was defined as the lowest concentration that completely inhibited visible growth.

### Pharmacokinetic study

LVFX (50, 100, and 200 mg/kg), GRNX (30, 60, and 120 mg/kg), and LSFX (10, 20, and 30 mg/kg) were administered as a single subcutaneous dose to the lung-infected mice. The dose range for each antimicrobial agent was selected to achieve a wide range of systemic exposures for PK/PD analysis, including subtherapeutic to therapeutically relevant levels, based on preliminary experiments and previous pharmacokinetic studies in mice (19, 20). Blood samples were collected from the inferior vena cava under general anesthesia at 5, 15, 30, 60, 120, 240, and 300 min after dosing, followed by euthanasia via exsanguination.

After thoracotomy, bronchoalveolar lavage fluid (BALF) was collected three times by intratracheal instillation and aspiration of sterile saline (0.5 mL). Blood and BALF samples were centrifuged, and the supernatants were collected as plasma and BALF, respectively.

All samples were subjected to protein precipitation before analysis. For LVFX and LSFX, methanol was added to the samples, followed by centrifugation at 15,000 × *g* for 10 min at 4°C. An aliquot of the supernatant was diluted with ultrapure water for high-performance liquid chromatography (HPLC) analysis. Acetonitrile was used as the precipitating solvent for GRNX, and the samples were processed under the same conditions. The concentrations of LVFX, GRNX, and LSFX were quantified using a validated HPLC method under the conditions described in Table 1, with accuracy and precision within ±5%. The lower limit of quantification (LLOQ) for each antimicrobial agent is provided in Table 1; all measured concentrations were above the LLOQ. The protein-binding ratio (%PB) of each antimicrobial agent in the mouse plasma was determined using ultrafiltration (21). Plasma samples spiked with each drug were incubated at 37°C for 30 min and then transferred to a Centrifree Ultrafiltration Device (Merck Millipore Ltd.). The samples were then centrifuged to obtain an ultrafiltrate. The drug concentrations in the total plasma before and after ultrafiltration were measured, and the %PB was calculated using the following equation:


$$\%PB=\left(1-\frac{C_{free}}{C_{total}}\right)*100$$

**TABLE 1:** HPLC conditions for the quantification of levofloxacin, garenoxacin, and lascufloxacin^*a*^

	Levofloxacin	Garenoxacin	Lascufloxacin
Column	Mightysil RP-18 GP 250 × 4.6 mm, 5 µm	Luna C18(2) 150 × 4.6 mm 5µm	CAPCELL PAK C_18_ MG II 5µm
Mobile phase	0.1% Phosphate buffer：MeOH = 16：9	0.2 M citrate buffer (pH 3.5): acetonitrile: water = 15:28:57	Acetic acid buffer (pH 3.5) : acetonitrile = 3:7
Detector	UV	Fluorescence	Fluorescence
Wavelength	294 nm	280/406 nm	289/498 nm
Column oven	25℃	30℃	40℃
Flow rate	0.5 mL/min	1.0 mL/min	1.2 mL/min
LLOQ	0.1 µg/mL	0.5 µg/mL	0.05 µg/mL

^*a*^ LLOQ, lower limit of quantification.

where C_free_ represents the drug concentration in the ultrafiltrate and C_total_ indicates the total drug concentration in the plasma before ultrafiltration.

Urea concentrations in the plasma and BALF were determined using a Urea Assay Kit (BioChain Institute, Inc., CA, USA). According to the manufacturer’s specifications, the assay has a linear detection range of 0.006–100 mg/dL (1 μM–17 mM) with a lower limit of detection of 0.006 mg/dL. All the measured samples were within this range and above the lower limit of detection. The drug concentrations in the ELF were calculated using the following equation (22):


$${Drug\;concentration}_{ELF}={Drug\;concentration}_{BALF}\times \left(\frac{{Urea\;concentration}_{plasma}}{{Urea\;concentration}_{BALF}}\right)$$

### Pharmacokinetic analysis

The concentration–time data for LVFX, GRNX, and LSFX in the plasma and ELF were analyzed using Phoenix WinNonlin (Certara, USA). PK analyses of the plasma and ELF concentrations were conducted separately. A one-compartment model with first-order absorption was fitted to the data using nonlinear least-squares regression, and the absorption rate constant (*k*_a_), volume of distribution (*V*_d_), and clearance (CL) were estimated. The model adequacy was evaluated using goodness-of-fit diagnostics, including observed-versus-predicted concentrations and residual-versus-time plots.

### *In vivo* pharmacodynamic study

Lung-infected mice were administered each antimicrobial agent subcutaneously using various dosing regimens 2–26 h after infection. Mice were euthanized by cervical dislocation 26 h after infection (or 2 h after infection for the control group), and lung tissues were aseptically excised.

Excised lungs were homogenized in saline using a Multi-Beads Shocker (Yasui Kikai, Osaka, Japan) at 1,800 rpm for 120 s at 18°C. The homogenates were subsequently serially diluted with saline. Undiluted and diluted samples were plated onto sheep blood agar plates and incubated overnight at 37°C in 5% CO_2_. Viable bacterial counts in the lungs were calculated as log_10_ CFU/lung.

### PK/PD analysis

Changes in bacterial counts obtained from the *in vivo* pharmacodynamic study were used as dependent variables, and their relationships with the PK/PD parameters (*f*T >MIC, *f*AUC/MIC, and *f*C_max_/MIC) for each dosing regimen were assessed. Analyses were performed using Phoenix WinNonlin with an inhibitory sigmoid *I*_max_ model. The model equation was as follows:


$$E=E_{0}-\left(\frac{I_{\max}*{PK/PD}_{index}^{\gamma }}{{PK/PD}_{index}^{\gamma }+{IC}_{50}^{\gamma }}\right)$$

where E represents the change in viable bacterial counts, E_0_ is the maximum bacterial burden, *I*_max_ is the maximum antibacterial effect, *PK/PD_index_* denotes the PK/PD parameter value, IC_50_ is the PK/PD parameter producing 50% of the maximum effect, and *ɤ* is the Hill coefficient describing the sigmoidicity of the curve.

In this study, the bactericidal endpoints of 1 log_10_ and 2 log_10_ reductions were selected as the primary PK/PD targets in accordance with the EMA PK/PD guidelines (23).

The following equation was used to translate murine ELF-based PK/PD targets into predicted human plasma targets:

Adjusted *f*AUC/MIC = (Target AUC_ELF_/MIC in the murine model) / (Human AUC_ELF_/*f*AUC_plasma_ ratio).

This approach is based on the assumption that PK/PD indices such as AUC/MIC are generally conserved across species and can be used for translational analyses of antimicrobial efficacy (24). In addition, the use of ELF-to-plasma exposure ratios allows the estimation of systemic drug exposure corresponding to target site concentrations (5).

## RESULTS

### MIC

The MICs of LVFX, GRNX, and LSFX against *S. pneumoniae* ATCC 49619 were 0.512, 0.032, and 0.032 mg/L, respectively. Both GRNX and LSFX exhibited lower MIC values than LVFX, indicating greater *in vitro* antibacterial activity.

### Plasma and ELF pharmacokinetics

Following a single subcutaneous administration of LVFX, GRNX, or LSFX to lung-infected mice, the plasma and ELF concentration–time profiles were characterized and analyzed using a one-compartment model. These diagnostic plots indicated no significant bias and supported the adequacy of the one-compartment model. The corresponding model evaluation results are presented in Fig. S1 to S6.

The PK parameters derived from the model analysis in plasma and ELF are summarized in Tables S1 to S3. The %PB was 15.5% ± 2.18 for LVFX, 88.6% ± 0.01 for GRNX, and 64.6% ± 1.06 for LSFX, respectively (mean ± SD).

### PK/PD analysis

**Fig 1:**
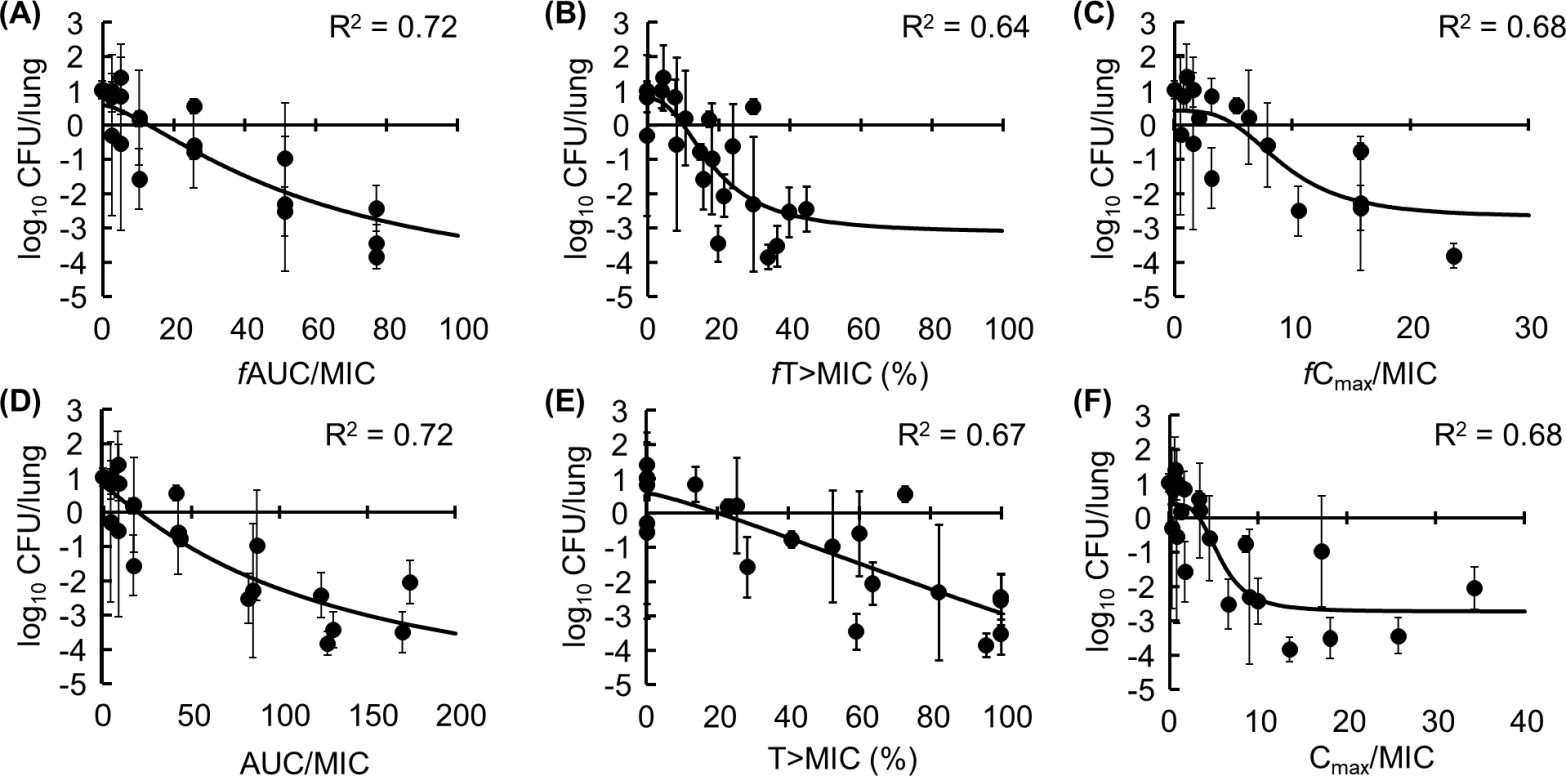
PK/PD analysis of levofloxacin in an *S. pneumoniae* murine lung infection model. Relationships between changes in pulmonary bacterial burden (log_10_ CFU/lung) and PK/PD indices are shown (*n* = 3, mean ± SD). Plasma-based PK/PD indices are shown for (**A**) *f*AUC/MIC, (**B**) *f*T>MIC, and (**C**) *f*C_max_/MIC. ELF-based PK/PD indices are shown for (**D**) AUC/MIC, (**E**) T>MIC, and (**F**) C_max_/MIC values.

**Fig 2:**
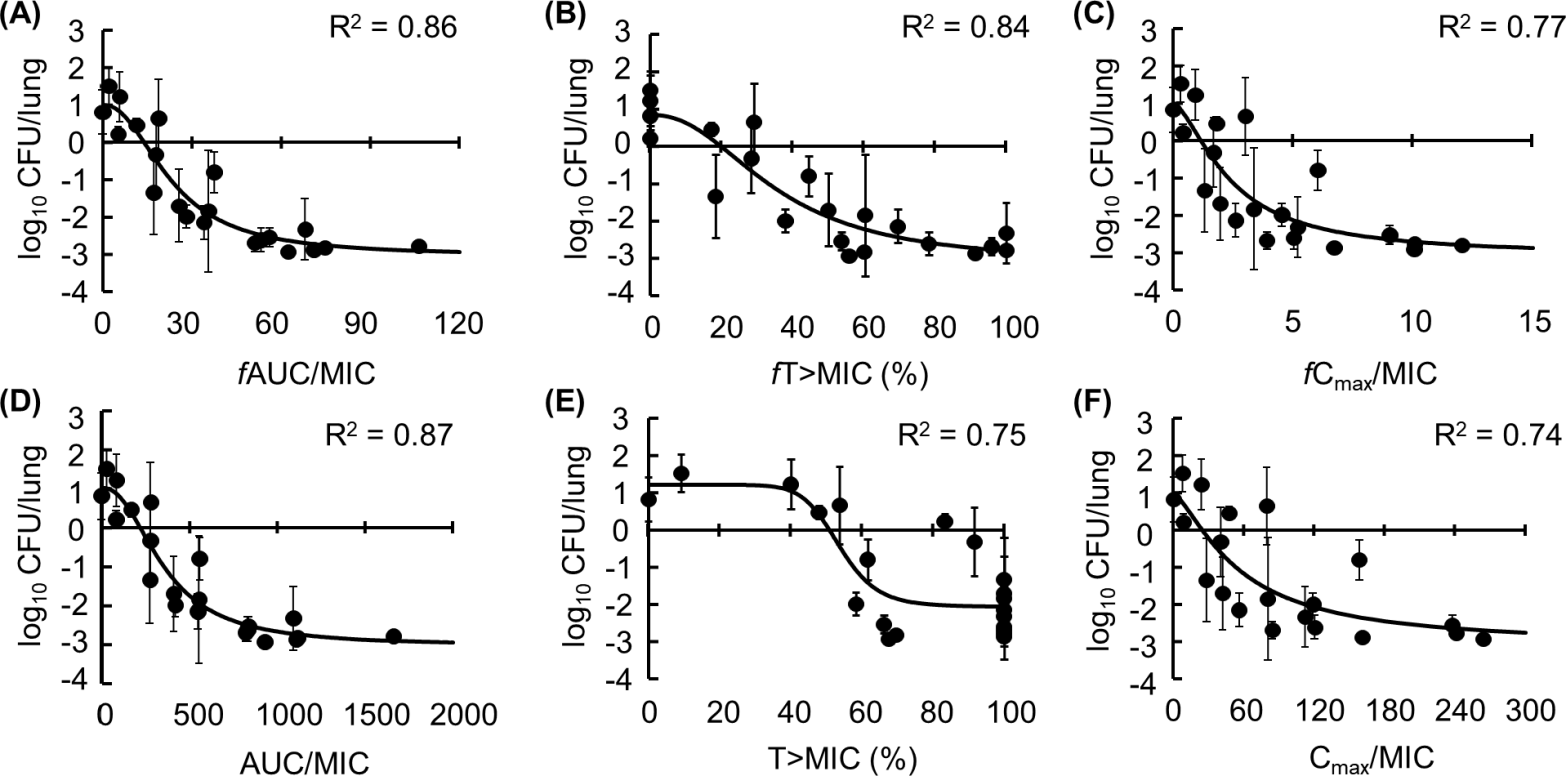
PK/PD analysis of garenoxacin in an *S. pneumoniae* murine lung infection model. Relationships between changes in pulmonary bacterial burden (log_10_ CFU/lung) and PK/PD indices are shown (*n* = 3, mean ± SD). Plasma-based PK/PD indices are shown for (**A**) *f*AUC/MIC, (**B**) *f*T>MIC, and (**C**) *f*C_max_/MIC. ELF-based PK/PD indices are shown for (**D**) AUC/MIC, (**E**) T>MIC, and (**F**) C_max_/MIC.

**Fig 3:**
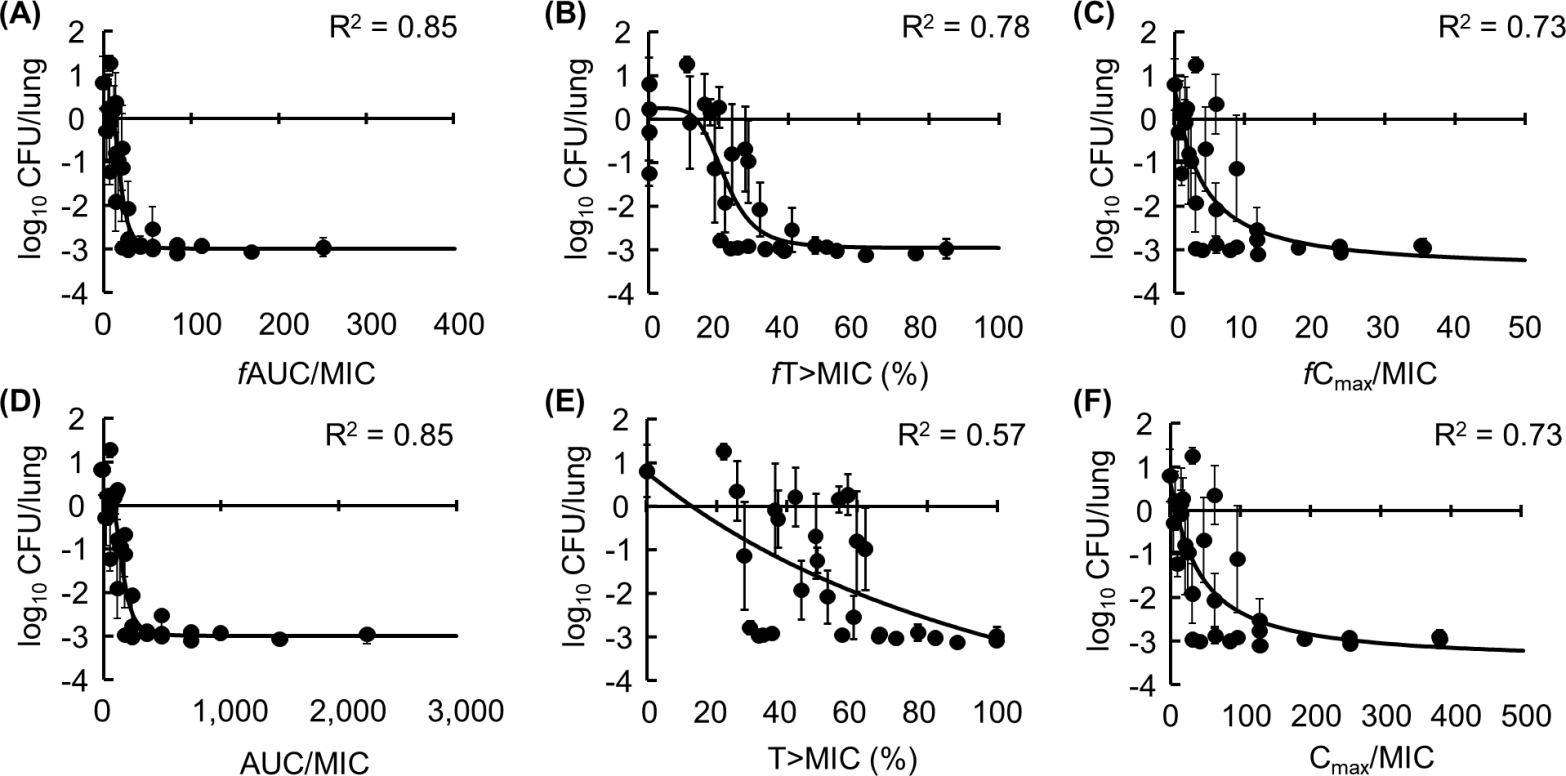
PK/PD analysis of lascufloxacin in an *S. pneumoniae* murine lung infection model. Relationships between changes in pulmonary bacterial burden (log_10_ CFU/lung) and PK/PD indices are shown (*n* = 3, mean ± SD). Plasma-based PK/PD indices are shown for (**A**) *f*AUC/MIC, (**B**) *f*T>MIC, and (**C**) *f*C_max_/MIC. ELF-based PK/PD indices are shown for (**D**) AUC/MIC, (**E**) T>MIC, and (**F**) C_max_/MIC.

For LVFX, GRNX, and LSFX, the relationships between the changes in bacterial counts and PK/PD indices were evaluated using a sigmoid *I*_max_ model (Fig. 1 to 3). Among the indices, *f*AUC/MIC based on the plasma concentrations showed the strongest correlation with the antibacterial effects of all three agents (R^2^ = 0.72 for LVFX, 0.86 for GRNX, and 0.85 for LSFX).

Based on the fitted models, the target *f*AUC_plasma_/MIC values required to achieve 1 log_10_ and 2 log_10_ bacterial killing were 13.1, 30.6, and 53.0 for LVFX; 13.8, 22.8, and 37.5 for GRNX; and 10.0, 16.5, and 22.8 for LSFX, respectively (Tables 2 to 4).

**TABLE 2:** PK/PD target values of levofloxacin in plasma and ELF

	*f*AUC/MIC	*f*T>MIC	*f*C_max_/MIC
PK/PD target values in plasma			
Stasis	13.1	10.2	5.2
1 log_10_ kill	30.6	17.1	8.7
2 log_10_ kill	53.0	27.1	13.6
PK/PD target values in ELF			
Stasis	19.5	20.5	3.4
1 log_10_ kill	48.6	47.9	5.4
2 log_10_ kill	88.6	74.9	8.0

**TABLE 3:** PK/PD target values of garenoxacin in plasma and ELF

	*f*AUC/MIC	*f*T>MIC	*f*C_max_/MIC
PK/PD target values in plasma			
Stasis	13.8	20.0	1.2
1 log_10_ kill	22.8	33.4	2.3
2 log_10_ kill	37.5	53.4	28.9
PK/PD target values in ELF			
Stasis	215.7	50.8	24.7
1 log_10_ kill	351.8	57.6	53.9
2 log_10_ kill	573.9	78.7	114.8

**TABLE 4:** PK/PD target values of lascufloxacin in plasma and ELF

	*f*AUC/MIC	*f*T>MIC	*f*C_max_/MIC
PK/PD target values in plasma			
Stasis	10.0	13.5	0.8
1 log_10_ kill	16.5	19.9	2.6
2 log_10_ kill	22.8	25.8	6.5
PK/PD target values in ELF			
Stasis	90.1	13.1	8.8
1 log_10_ kill	147.7	33.6	28.1
2 log_10_ kill	204.0	60.8	70.1

PK/PD analyses based on ELF concentrations also identified AUC/MIC as the index most strongly correlated with antibacterial effects (R^2^ = 0.72–0.87). The AUC_ELF_/MIC targets for the static effect, 1 log_10_, and 2 log_10_ killing were 19.5, 48.6, and 88.6 for LVFX; 215.7, 351.8, and 573.9 for GRNX; and 90.1, 147.7, and 204.0 for LSFX, respectively (Tables 2 to 4).

### Prediction of clinical PK/PD targets based on PK/PD analysis

The reported ELF penetration ratios (AUC_ELF_/*f*AUC_plasma_) of LVFX, GRNX, and LSFX in humans were 1.16, 5.38, and 61.7, respectively (1, 16, 25). Using these ratios, the murine AUC_ELF_/MIC targets required to achieve 1 log_10_ and 2 log_10_ killing in the murine lung infection model were converted into the corresponding human *f*AUC_plasma_/MIC targets. The predicted human target *f*AUC_plasma_/MIC values required to achieve 1 log_10_ killing were 41.9 for LVFX, 65.4 for GRNX, and 2.39 for LSFX, whereas those required to achieve 2 log_10_ killing were 76.4, 106.7, and 3.31, respectively (Table 5).

**TABLE 5:** Target *f*AUC/MIC values derived from plasma- and ELF-based PK/PD analyses with adjustment using human ELF penetration ratios

Antimicrobial agent		Target *f*AUC/MIC (mouse PK/PD model)		Adjusted target *f*AUC/MIC considering human ELF penetration^*a*^	Clinical target *f*AUC/MIC (literature)
		In plasma	In ELF		
Levofloxacin	1 log_10_ kill	30.6	48.6	48.6/1.16^[25]^ = 41.9	> 30 ^[3]^
	2 log_10_ kill	53.0	88.6	88.6/1.16^[25]^ = 76.4	
Garenoxacin	1 log_10_ kill	22.8	351.8	351.8/5.38^[1]^ = 65.4	> 50 ^[4]^
	2 log_10_ kill	37.5	573.9	573.9/5.38^[1]^ = 106.7	
Lascufloxacin	1 log_10_ kill	16.5	147.7	147.7/61.7^[16]^ = 2.39	> 3.9 ^[5]^
	2 log_10_ kill	22.8	204.0	204.0/61.7^[16]^ = 3.31	

^*a*^ Adjusted *f*AUC/MIC = (Target AUC_ELF_/MIC in mouse model) / (Human AUC_ELF_/*f*AUC_Plasma_ ratio).

## DISCUSSION

In this study, we aimed to clarify the effect of infection site–specific drug exposure on the interpretation of PK/PD targets in pulmonary infections by directly comparing three fluoroquinolones with different ELF penetrations under identical experimental conditions in a murine lung infection model. Our results demonstrate that site-specific drug exposure substantially influences the interpretation of PK/PD targets and, consequently, the assessment of antibacterial efficacy in lung infections.

In plasma-based PK/PD analyses, *f*AUC_plasma_/MIC showed the strongest correlation with the antibacterial effects of all three agents, which is consistent with the established PK/PD principles for fluoroquinolones (6–8). Importantly, ELF-based analyses demonstrated that the AUC_ELF_/MIC also represented the primary PK/PD index across all agents. This finding indicates that the PK/PD driver of fluoroquinolone efficacy remains consistent, even when evaluated at the pulmonary site of infection.

ELF-based PK/PD targets derived from a murine lung infection model were translated into the corresponding human *f*AUC_plasma_/MIC targets using the reported human ELF-to-plasma exposure ratios (AUC_ELF_/*f*AUC_plasma_). The predicted human targets were generally consistent with the clinically established PK/PD targets, supporting the overall validity of this translational approach.

Comparative analyses revealed that differences in ELF penetration among the three agents influenced the PK/PD targets. LVFX exhibited relatively low ELF penetration, and ELF-based analysis did not substantially change the PK/PD target compared with the plasma-based analysis. GRNX exhibited higher ELF penetration than LVFX in the murine model and achieved greater drug exposure at the infection site; however, the incorporation of ELF exposure did not markedly alter PK/PD target interpretation compared with plasma-based analysis. In contrast, LSFX demonstrated higher drug exposure in ELF than in plasma, resulting in a clear divergence between the PK/PD targets estimated from the ELF- and plasma-based analyses. The target estimates differed from those derived from plasma-based analyses when PK/PD relationships were evaluated based on ELF exposure, indicating that site-specific drug exposure contributed substantially to antibacterial efficacy. Furthermore, when ELF-based PK/PD targets obtained from the murine lung infection model were translated into predicted human *f*AUC_plasma_/MIC values using the reported ELF-to-plasma exposure ratios, the resulting estimates were generally consistent with those of the clinically established PK/PD targets. These findings indicate that ELF-based PK/PD evaluation provides important information for interpreting antibacterial efficacy, particularly for agents with a substantial pulmonary distribution. Considering the high ELF penetration reported in humans, LSFX may achieve high drug exposure at the pulmonary infection site. However, the clinical relevance of the predicted PK/PD targets requires careful interpretation, particularly given potential interspecies differences in pulmonary drug distribution. Notably, despite similar MIC values between GRNX and LSFX, distinct ELF-based AUC/MIC targets were identified, indicating that pulmonary distribution within the same antimicrobial class can substantially influence PK/PD target magnitude.

The findings of this study have practical implications for antimicrobial selection and dose optimization for treating pulmonary infections. For agents with high pulmonary penetration, reliance solely on plasma-based PK/PD indices may underestimate effective drug exposure at the infection site. Therefore, the incorporation of ELF-based PK/PD analysis may improve the prediction of antibacterial efficacy. Similar considerations emphasizing infection-site pharmacokinetics have been highlighted in translational infection models such as hollow-fiber systems. Although this study does not directly compare clinical outcomes, the present findings suggest that respiratory fluoroquinolones may not be fully interchangeable as a class when pulmonary drug distribution differs substantially.

This study has several limitations. When ELF concentrations are assessed using BALF, the recovered BALF represents a mixture of ELF and instilled saline, thereby reflecting an apparent concentration rather than the true ELF concentration. Rennard et al. demonstrated, using the urea dilution method, that only a small volume of ELF is recovered from BALF and that the degree of dilution and recovered concentrations can vary substantially depending on the procedure (22). Accordingly, although ELF-based PK/PD indices are useful for reflecting pulmonary site-specific pharmacokinetics, their interpretation should consider the dilution bias inherent in the BALF method. In addition, the human PK/PD targets presented in this study were extrapolated from the PK/PD relationships identified in a murine lung infection model and should be interpreted with caution in light of interspecies differences in pulmonary drug distribution. In addition, humanized pharmacokinetic profiles were not evaluated in this study, which may limit its direct clinical translation. Furthermore, only a single strain of *S. pneumoniae*, lacking relevant resistance mechanisms, was evaluated, potentially limiting the generalizability of the findings. Evaluation of multiple strains with diverse susceptibility profiles is generally recommended in PK/PD studies. Finally, drug and urea concentrations were quantified using HPLC-based and colorimetric assays rather than LC-MS/MS, which may have affected the analytical sensitivity and specificity.

### Conclusion

This study demonstrates the importance of site-specific drug exposure in the ELF for evaluating PK/PD relationships in pulmonary infections. By directly comparing the three fluoroquinolones under identical experimental conditions, we showed that differences in intrapulmonary drug distribution substantially influenced PK/PD targets and that plasma-based indices alone may not adequately capture antibacterial efficacy in the lungs. These findings support the integration of ELF-based PK/PD evaluation into antimicrobial selection and dose optimization strategies for treating pulmonary infections.

## SUPPLEMENTAL MATERIAL

Supplemental figures aac.00375-26-s0001.docx Fig. S1 to S6.

Supplemental tables aac.00375-26-s0002.docx Tables S1 to S6.

ASM does not own the copyrights to Supplemental Material that may be linked to, or accessed through, an article. The authors have granted ASM a non-exclusive, world-wide license to publish the Supplemental Material files. Please contact the corresponding author directly for reuse.
