# Supplementary material for: Three fluoroquinolones with different epithelial lining fluid penetration in a murine lung infection model: an experimental site-specific PK/PD study
Source: Antimicrob Agents Chemother. 2026 Jun 22;70(8):e00375-26. doi: 10.1128/aac.00375-26 (PMC13435811; doi:10.1128/aac.00375-26)
Supplement: Supplementary file 1 [file aac.00375-26-s0001.docx]

**Supplementary Figure S1 Observed vs predicted concentrations for levofloxacin.**

Observed concentrations plotted against model‑predicted concentrations for levofloxacin: (A) Plasma 50 mg/kg, (B) Plasma 100 mg/kg, (C) Plasma 200 mg/kg, (D) ELF 50 mg/kg, (E) ELF 100 mg/kg, (F) ELF 200 mg/kg.

**Supplementary Figure S2 Residuals vs time for levofloxacin.**

Residuals plotted against time after dosing for levofloxacin: (A) Plasma 50 mg/kg, (B) Plasma 100 mg/kg, (C) Plasma 200 mg/kg, (D) ELF 50 mg/kg, (E) ELF 100 mg/kg, (F) ELF 200 mg/kg.

**Supplementary Figure S3 Observed vs predicted concentrations for garenoxacin.**

Observed concentrations plotted against model‑predicted concentrations for garenoxacin: (A) Plasma 30 mg/kg, (B) Plasma 60 mg/kg, (C) Plasma 120 mg/kg, (D) ELF 30 mg/kg, (E) ELF 60 mg/kg, (F) ELF 120 mg/kg.

**Supplementary Figure S4 Residuals vs time for garenoxacin.**

Residuals plotted against time after dosing for garenoxacin: (A) Plasma 30 mg/kg, (B) Plasma 60 mg/kg, (C) Plasma 120 mg/kg, (D) ELF 30 mg/kg, (E) ELF 60 mg/kg, (F) ELF 120 mg/kg.

**Supplementary Figure S5 Observed vs predicted concentrations for lascufloxacin.**

Observed concentrations plotted against model‑predicted concentrations for lascufloxacin: (A) Plasma 10 mg/kg, (B) Plasma 20 mg/kg, (C) Plasma 30 mg/kg, (D) ELF 10 mg/kg, (E) ELF 20 mg/kg, (F) ELF 30 mg/kg.

**Supplementary Figure S6 Residuals vs time for lascufloxacin.**

Residuals plotted against time after dosing for lascufloxacin: (A) Plasma 10 mg/kg, (B) Plasma 20 mg/kg, (C) Plasma 30 mg/kg, (D) ELF 10 mg/kg, (E) ELF 20 mg/kg, (F) ELF 30 mg/kg.
