# Supplementary material for: Three fluoroquinolones with different epithelial lining fluid penetration in a murine lung infection model: an experimental site-specific PK/PD study
Source: Antimicrob Agents Chemother. 2026 Jun 22;70(8):e00375-26. doi: 10.1128/aac.00375-26 (PMC13435811; doi:10.1128/aac.00375-26)
Supplement: Supplementary file 2 [file aac.00375-26-s0002.docx]

**Supplementary Table 1 Pharmacokinetic parameter estimates of levofloxacin in plasma and ELF**

| Dose (mg/kg) | Plasma | | |  | ELF | | |
| --- | --- | --- | --- | --- | --- | --- | --- |
|  | *V*_d_ (L/kg) | *k*_a_ (1/h) | CL(L/h/kg) |  | *V*_d_ (L/kg) | *k*_a_ (1/h) | CL(L/h/kg) |
| 50 | 4.46 | 7.10 | 4.04 |  | 1.75 | 7.27 | 2.95 |
| 100 | 4.60 | 3.54 | 4.33 |  | 2.65 | 12.67 | 1.93 |
| 200 | 4.86 | 1.80 | 3.00 |  | 3.39 | 6.56 | 1.82 |
| Mean | 4.64 | 4.14 | 3.79 |  | 2.60 | 8.83 | 2.24 |
| SD | 0.17 | 2.21 | 0.57 |  | 0.67 | 2.73 | 0.51 |

*V*_d_, distribution volume；*k*_a_, absorption rate constant；CL, clearance.

**Supplementary Table 2 Pharmacokinetic parameter estimates of garenoxacin in plasma and ELF**

| Dose (mg/kg) | Plasma | | |  | ELF | | |
| --- | --- | --- | --- | --- | --- | --- | --- |
|  | *V*_d_ (L/kg) | *k*_a_ (1/h) | CL(L/h/kg) |  | *V*_d_ (L/kg) | *k*_a_ (1/h) | CL(L/h/kg) |
| 30 | 42.88 | 5.36 | 4.67 |  | 4.89 | 1.30 | 2.26 |
| 60 | 19.48 | 4.06 | 3.99 |  | 13.62 | 2.79 | 4.89 |
| 120 | 28.34 | 1.70 | 8.31 |  | 9.06 | 7.14 | 2.92 |
| Mean | 30.23 | 3.71 | 5.65 |  | 9.19 | 3.74 | 3.36 |
| SD | 3.72 | 0.94 | 0.98 |  | 1.92 | 1.71 | 0.63 |

*V*_d_, distribution volume；*k*_a_, absorption rate constant；CL, clearance.

**Supplementary Table 3 Pharmacokinetic parameter estimates of lascufloxacin in plasma and ELF**

| Dose (mg/kg) | Plasma | | |  | ELF | | |
| --- | --- | --- | --- | --- | --- | --- | --- |
|  | *V*_d_ (L/kg) | *k*_a_ (1/h) | CL(L/h/kg) |  | *V*_d_ (L/kg) | *k*_a_ (1/h) | CL(L/h/kg) |
| 10 | 4.00 | 3.17 | 3.88 |  | 1.22 | 2.10 | 1.08 |
| 20 | 5.13 | 1.43 | 4.65 |  | 1.15 | 1.19 | 1.35 |
| 30 | 10.56 | 6.06 | 3.35 |  | 2.53 | 8.00 | 1.32 |
| Mean | 6.56 | 3.55 | 3.96 |  | 1.63 | 3.76 | 1.25 |
| SD | 2.87 | 1.91 | 0.53 |  | 0.63 | 3.02 | 0.12 |

*V*_d_, distribution volume；*k*_a_, absorption rate constant；CL, clearance.

**Supplementary Table 4 PK/PD sigmoid *I*_max_ model parameter estimates of levofloxacin based on plasma and ELF concentrations**

|  | *f*AUC/MIC | *f*T>MIC | *f*C_max_/MIC |
| --- | --- | --- | --- |
| Plasma | | | |
| *I*_max_ | 5.61 | 3.93 | 3.12 |
| IC_50_ | 58.59 | 18.43 | 9.18 |
| E_0_ | 0.61 | 0.80 | 0.43 |
| *ɤ* | 1.41 | 2.33 | 3.21 |
| ELF | | | |
| *I*_max_ | 6.82 | 17.73 | 3.11 |
| IC_50_ | 125.08 | 313.04 | 55.71 |
| E_0_ | 0.76 | 0.60 | 0.40 |
| *ɤ* | 1.12 | 1.23 | 3.63 |

**Supplementary Table 5 PK/PD sigmoid *I*_max_ model parameter estimates of garenoxacin based on plasma and ELF concentrations**

|  | *f*AUC/MIC | *f*T>MIC | *f*C_max_/MIC |
| --- | --- | --- | --- |
| Plasma | | | |
| *I*_max_ | 4.05 | 4.00 | 4.07 |
| IC_50_ | 22.91 | 35.50 | 2.37 |
| E_0_ | 1.02 | 0.86 | 0.99 |
| *ɤ* | 2.17 | 2.25 | 1.58 |
| ELF | | | |
| *I*_max_ | 4.05 | 3.29 | 4.04 |
| IC_50_ | 351.50 | 53.53 | 55.75 |
| E_0_ | 1.03 | 1.22 | 0.97 |
| *ɤ* | 2.21 | 10.00 | 1.41 |

**Supplementary Table 6 PK/PD sigmoid *I*_max_ model parameter estimates of lascufloxacin based on plasma and ELF concentrations**

|  | *f*AUC/MIC | *f*T>MIC | *f*C_max_/MIC |
| --- | --- | --- | --- |
| Plasma | | | |
| *I*_max_ | 3.27 | 3.21 | 4.14 |
| IC_50_ | 18.47 | 21.70 | 3.77 |
| E_0_ | 0.27 | 0.26 | 0.66 |
| *ɤ* | 3.93 | 5.09 | 1.09 |
| ELF | | | |
| *I*_max_ | 3.27 | 8.71 | 4.15 |
| IC_50_ | 165.57 | 128.02 | 40.60 |
| E_0_ | 0.27 | 0.78 | 0.67 |
| *ɤ* | 3.94 | 1.02 | 1.08 |
